# Crystal structure of bis[4-(di­methyl­amino)­pyridinium] bis(2-nitro­benzoate) trihydrate

**DOI:** 10.1107/S1600536814020583

**Published:** 2014-09-20

**Authors:** N. Sivakumar, S. Muralidharan, G. Chakkaravarthi, D. Velmurugan, G. Anbalagan

**Affiliations:** a Department of Physics, Presidency College, Chennai 600 005, India; b Department of Physics, Anna University, Chennai 600 025, India; c Department of Physics, CPCL Polytechnic College, Chennai 600 068, India; d Centre of Advanced Study in Crystallography and Biophysics, University of Madras, Chennai 600 025, India

**Keywords:** crystal structure, 4-di­methyl­amino­pyrdidine, 2-nitro­benzoic acid, salt, hydrogen bonds, π–π inter­actions, C—H⋯π inter­actions

## Abstract

The title salt, 2C_7_H_11_N_2_^+^·2C_7_H_4_NO_4_^−^·3H_2_O, crystallized with two anions and two cations in the asymmetric unit, together with three water mol­ecules. Both 4-di­methyl­amino­pyridinium cations are protonated at their pyridine N atoms with the plane of the N(CH_3_)_2_ hetero atoms inclined to the pyridine ring by 4.5 (2) and 1.4 (2)°. In the 2-nitro­benzoate anions, the carboxyl and nitro groups are inclined to their respective benzene rings by 77.1 (3) and 20.0 (3)°, and 75.8 (2) and 20.9 (3)°. In the crystal, the anions are linked *via* O—H⋯O hydrogen bonds involving the water mol­ecules, forming chains along [100]. The cations are linked to these chains by N—H⋯O hydrogen bonds. The chains are linked *via* C—H⋯O hydrogen bonds and C—H⋯π and π–π inter­actions [inter-centroid distances range from 3.617 (1) to 3.851 (1) Å], forming a three-dimensional structure.

## Abstract 2

The title salt crystallized with two anions and two cations in the asymmetric unit, together with three water mol­ecules. In the crystal, the anions are linked *via* O—H⋯O hydrogen bonds, involving the water mol­ecules, forming chains along [100] and the cations are linked to these chains by N—H⋯O hydrogen bonds.

## Chemical context   

Pyridine derivatives are used as calcium channel blockers and antagonists, and exhibit biological activities such as fungicidal, anti­bacterial, anti­fungal, anti­mycotic (Bossert *et al.*, 1981 ▶; Lohaus & Dittmar, 1968 ▶; Wang *et al.*,1989 ▶). Benzene derivatives are extensively used in medicinal chemistry as important inter­mediates for many pharmaceutical products (Altmann *et al.*, 2004 ▶). We herein report on the synthesis and crystal structure of the title salt prepared by the reaction of 4-di­methyl­amino­pyridine with 2-nitro­benzoic acid in hot ethanol as solvent.
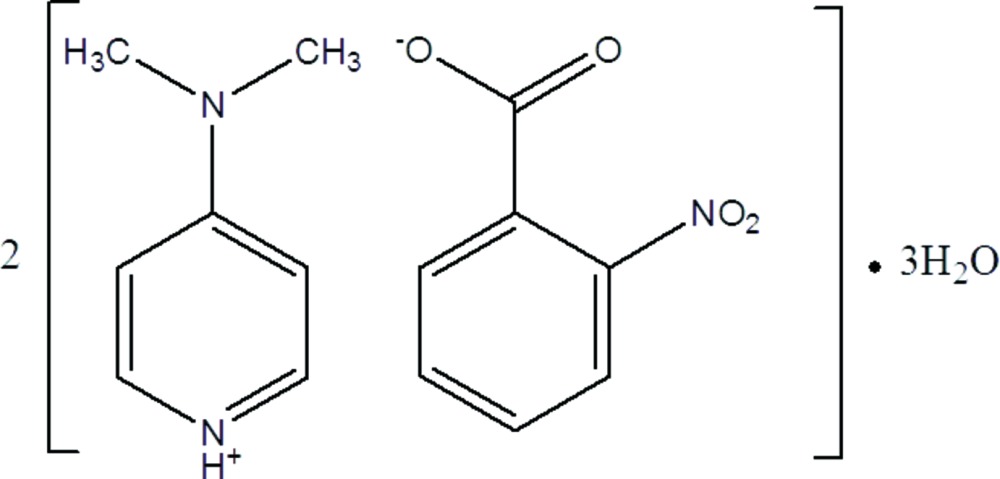


## Structural commentary   

**Figure 1:**
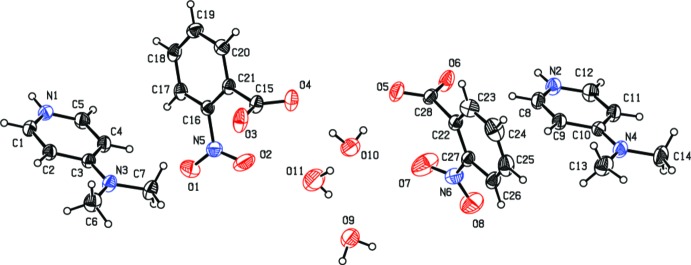
The mol­ecular structure of the title salt, with atom labelling. Displacement ellipsoids are drawn at the 30% probability level.

The asymmetric unit of the title salt consists of two 4-di­methyl­amino­pyrdinium cations and two 2-nitro­benzoate anions, together with three water mol­ecules (Fig. 1 ▶). The geometric parameters of the title compound are comparable to those reported for similar structures (Babu *et al.*, 2014 ▶; Rajkumar *et al.*, 2014 ▶), including the compounds 4-di­methyl­amino­pyridinium 2,4-, 3,4- and 3,5-di­nitro­benzoate (Hosomi *et al.*, 2000 ▶). The conformations of the two cations are very similar as are the conformations of the two anions. Both 4-di­methyl­amino­pyridinium cations are protonated at their pyridine N atoms (N1 and N2) with the planes of the N(CH_3_)_2_ hetero atoms (N3/C6/C7 and N4/C13/C14) inclined to the pyridine rings (N1/C1–C5 and N2/C8–C12) by 4.5 (2) and 1.4 (2)°, respectively. In the 2-nitro­benzoate anions the carboxyl groups (O3/O4/C15 and O5/O6/C28) are inclined to the respective benzene rings (C16–C21 and C22–C27) by 77.1 (3) and 75.8 (2)°. The nitro groups (O1/O2/N5 and O7/O8/N6) are inclined to their respective benzene rings by 20.0 (3) and 20.9 (3)°.

## Supra­molecular features   

**Figure 2:**
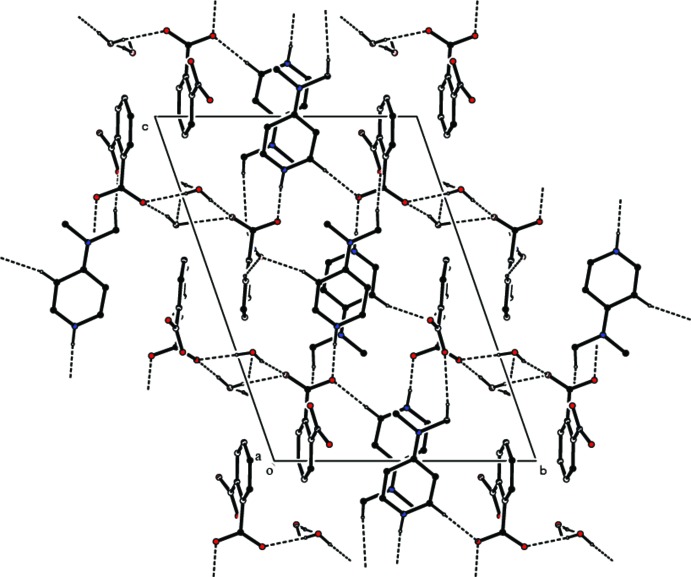
A view along the *a* axis of the crystal packing of the title salt. Hydrogen bonds are shown as dashed lines (see Table 1 ▶ for details; H atoms not involved in hydrogen bonding have been omitted for clarity).

In the crystal, the dihedral angle between the two pyridine rings (N1/C1–C5 and N2/C8–C12) is 5.16 (9)°, while the benzene rings (C16–C21 and C22–C27) form a dihedral angle of 19.56 (9)°. The anions are linked *via* O—H⋯O hydrogen bonds involving the water mol­ecules, forming chains along [100]; Table 1 ▶ and Fig. 2 ▶. The cations are linked to these chains by N—H⋯O hydrogen bonds (Table 1 ▶). The chains are linked *via* C—H⋯O hydrogen bonds and C—H⋯π and π–π inter­actions, forming a three-dimensional structure [*Cg*1⋯*Cg*1^i^ = 3.851 (1); *Cg*2⋯*Cg*2^ii^ = 3.656 (1); *Cg*3⋯*Cg*3^iii^ = 3.617 (1) Å; *Cg*1, *Cg*2, and *Cg*3 are the centroids of rings N1/C1–C5, N2/C8–C12, and C16–C21, respectively; symmetry codes: (i) −*x* + 2, −*y* + 1, −*z* + 2; (ii) −*x* + 2, −*y* + 3, −*z* + 1; (iii) −*x* + 2, −*y* + 2, −*z* + 2].

## Database survey   

A search of the Cambridge Structural Database (Version 5.35, last update May 2014; Allen, 2002 ▶) of salts including benzoate anions and the cation 4-di­methyl­amino­pyridinium yielded 15 hits. Three of these salts have as anions 2,4-di­nitro­benzoate, 3,4-di­nitro­benzoate and 3,5-di­nitro­benzoate (KOBMAP, KOBNAQ, and KOBNOE, respectively; Hosomi *et al.*, 2000 ▶). They were studied for their potential SHG properties; only the 3,5-dintrobenzoate salt crystallized in a non-centrosymmetric space group.

## Synthesis and crystallization   

4-Di­methyl­amino­pyridine (2.442 g, 1 mmol) and 2-nitro­benzoic acid (3.342 g, 1mmol) were dissolved in 50 ml of hot ethanol as a solvent. The mixture was stirred well for 8 h to give a homogeneous solution and is was then allowed to evaporate in air at room temperature. Within a few days, small colourless block-like crystals of the title salt were formed.

## Refinement   

Crystal data, data collection and structure refinement details are summarized in Table 2 ▶. Water H atoms and the pyridinium N atoms were located from difference Fourier maps and refined with distance restraints. O—H = 0.82 (1) and N—H = 0.88 (1) Å, with *U*_iso_(H) = 1.5*U*_eq_(O) for the water H atoms. The C-bound H atoms were positioned geometrically and refined using a riding model: C—H = 0.93–0.96 Å with *U*_iso_(H) = 1.5*U*_eq_(C) for methyl H atoms and = 1.2*U*_eq_(C) for other H atoms.

## Supplementary Material

Crystal structure: contains datablock(s) I, global. DOI: 10.1107/S1600536814020583/su2777sup1.cif

Structure factors: contains datablock(s) I. DOI: 10.1107/S1600536814020583/su2777Isup2.hkl

Supporting information file. DOI: 10.1107/S1600536814020583/su2777Isup3.cml

CCDC reference: 1024182

Additional supporting information:  crystallographic information; 3D view; checkCIF report

## Figures and Tables

**Table 1:** Hydrogen-bond geometry (Å, °) *Cg*1, *Cg*3 and *Cg*4 are the centroids of rings N1/C1–C5, C16–C21, and C22–C27, respectively.

*D*—H⋯*A*	*D*—H	H⋯*A*	*D*⋯*A*	*D*—H⋯*A*
N1—H1*A*⋯O6^i^	0.89 (2)	1.75 (2)	2.639 (2)	175 (2)
O9—H9*A*⋯O5^ii^	0.84 (4)	2.11 (4)	2.896 (2)	156 (4)
O10—H10*A*⋯O4	0.82 (4)	2.08 (4)	2.892 (2)	170 (4)
O10—H10*B*⋯O5	0.82 (3)	2.02 (3)	2.847 (3)	176 (4)
O11—H11*A*⋯O9	0.83 (4)	2.05 (3)	2.841 (3)	160 (4)
O11—H11*B*⋯O10	0.86 (4)	2.12 (4)	2.943 (3)	160 (4)
C1—H1⋯O3^iii^	0.93	2.43	3.348 (2)	169
C4—H4⋯O1	0.93	2.57	3.474 (3)	164
C6—H6*B*⋯O7^iv^	0.96	2.51	3.258 (3)	135
C8—H8⋯O6	0.93	2.40	3.325 (3)	177
C23—H23⋯O8^v^	0.93	2.55	3.434 (3)	160
C2—H2⋯*Cg*3^iii^	0.93	2.96	3.7481 (19)	144
C7—H7*A*⋯*Cg*1^vi^	0.93	2.85	3.661 (2)	142
C9—H9⋯*Cg*4	0.93	2.86	3.702 (2)	151

Symmetry codes: (i) 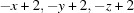; (ii) 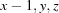; (iii) 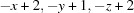; (iv) 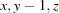; (v) 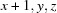; (vi) 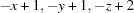.

**Table 2:** Experimental details

Crystal data	
Chemical formula	2C_7_H_11_N_2_^+^·2C_7_H_4_NO_4_^−^·3H_2_O
*M*_r_	632.63
Crystal system, space group	Triclinic, *P* 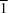
Temperature (K)	296
*a*, *b*, *c* (Å)	7.6163 (4), 12.7215 (7), 17.3478 (9)
α, β, γ (°)	108.238 (1), 92.247 (2), 101.512 (1)
*V* (Å^3^)	1554.93 (14)
*Z*	2
Radiation type	Mo *K*α
μ (mm^−1^)	0.11
Crystal size (mm)	0.28 × 0.24 × 0.20

Data collection	
Diffractometer	Bruker APEXII CCD
Absorption correction	Multi-scan (*SADABS*; Sheldrick, 1996 ▶)
*T*_min_, *T*_max_	0.971, 0.979
No. of measured, independent and observed [*I* > 2σ(*I*)] reflections	24031, 6426, 5126
*R*_int_	0.029
(sin θ/λ)_max_ (Å^−1^)	0.627

Refinement	
*R*[*F* ^2^ > 2σ(*F* ^2^)], *wR*(*F* ^2^), *S*	0.049, 0.154, 1.04
No. of reflections	6426
No. of parameters	437
No. of restraints	9
H-atom treatment	H atoms treated by a mixture of independent and constrained refinement
Δρ_max_, Δρ_min_ (e Å^−3^)	0.33, −0.36

Computer programs: *APEX2* and *SAINT* (Bruker, 2004 ▶), *SHELXS97* and *SHELXL97* (Sheldrick, 2008 ▶) and *PLATON* (Spek, 2009 ▶).

## supplementary crystallographic information

### Crystal data

**Table:**

2C_7_H_11_N_2_^+^·2C_7_H_4_NO_4_^−^·3H_2_O	*Z* = 2
*M**_r_* = 632.63	*F*(000) = 668
Triclinic, *P*1	*D*_x_ = 1.351 Mg m^−^^3^
Hall symbol: -P 1	Mo *K*α radiation, λ = 0.71073 Å
*a* = 7.6163 (4) Å	Cell parameters from 668 reflections
*b* = 12.7215 (7) Å	θ = 1.2–26.5°
*c* = 17.3478 (9) Å	µ = 0.11 mm^−^^1^
α = 108.238 (1)°	*T* = 296 K
β = 92.247 (2)°	Block, colourless
γ = 101.512 (1)°	0.28 × 0.24 × 0.20 mm
*V* = 1554.93 (14) Å^3^	

### Data collection

**Table:**

Bruker APEXII CCD diffractometer	6426 independent reflections
Radiation source: fine-focus sealed tube	5126 reflections with *I* > 2σ(*I*)
Graphite monochromator	*R*_int_ = 0.029
ω and φ scan	θ_max_ = 26.5°, θ_min_ = 1.2°
Absorption correction: multi-scan (*SADABS*; Sheldrick, 1996)	*h* = −9→9
*T*_min_ = 0.971, *T*_max_ = 0.979	*k* = −15→15
24031 measured reflections	*l* = −21→21

### Refinement

**Table:**

Refinement on *F*^2^	Secondary atom site location: difference Fourier map
Least-squares matrix: full	Hydrogen site location: inferred from neighbouring sites
*R*[*F*^2^ > 2σ(*F*^2^)] = 0.049	H atoms treated by a mixture of independent and constrained refinement
*wR*(*F*^2^) = 0.154	*w* = 1/[σ^2^(*F*_o_^2^) + (0.074*P*)^2^ + 0.5049*P*] where *P* = (*F*_o_^2^ + 2*F*_c_^2^)/3
*S* = 1.04	(Δ/σ)_max_ < 0.001
6426 reflections	Δρ_max_ = 0.33 e Å^−^^3^
437 parameters	Δρ_min_ = −0.36 e Å^−^^3^
9 restraints	Extinction correction: *SHELXL*, Fc^*^=kFc[1+0.001xFc^2^λ^3^/sin(2θ)]^-1/4^
Primary atom site location: structure-invariant direct methods	Extinction coefficient: 0.088 (5)

### Special details

**Table:**

Geometry. All e.s.d.'s (except the e.s.d. in the dihedral angle between two l.s. planes) are estimated using the full covariance matrix. The cell e.s.d.'s are taken into account individually in the estimation of e.s.d.'s in distances, angles and torsion angles; correlations between e.s.d.'s in cell parameters are only used when they are defined by crystal symmetry. An approximate (isotropic) treatment of cell e.s.d.'s is used for estimating e.s.d.'s involving l.s. planes.
Refinement. Refinement of *F*^2^ against ALL reflections. The weighted *R*-factor *wR* and goodness of fit *S* are based on *F*^2^, conventional *R*-factors *R* are based on *F*, with *F* set to zero for negative *F*^2^. The threshold expression of *F*^2^ > σ(*F*^2^) is used only for calculating *R*-factors(gt) *etc*. and is not relevant to the choice of reflections for refinement. *R*-factors based on *F*^2^ are statistically about twice as large as those based on *F*, and *R*- factors based on ALL data will be even larger.

### Fractional atomic coordinates and isotropic or equivalent isotropic displacement parameters (Å^2^)

**Table:**

	*x*	*y*	*z*	*U*_iso_*/*U*_eq_	
C1	0.8708 (2)	0.46302 (15)	1.12324 (10)	0.0488 (4)	
H1	0.9041	0.4227	1.1556	0.059*	
C2	0.7934 (2)	0.40558 (14)	1.04589 (10)	0.0460 (4)	
H2	0.7728	0.3268	1.0263	0.055*	
C3	0.7434 (2)	0.46462 (13)	0.99459 (9)	0.0409 (3)	
C4	0.7752 (2)	0.58414 (14)	1.03061 (11)	0.0483 (4)	
H4	0.7426	0.6276	1.0005	0.058*	
C5	0.8525 (3)	0.63547 (15)	1.10852 (11)	0.0527 (4)	
H5	0.8727	0.7141	1.1309	0.063*	
C6	0.6297 (3)	0.28730 (17)	0.88249 (12)	0.0664 (5)	
H6A	0.7376	0.2625	0.8660	0.100*	
H6B	0.5412	0.2632	0.8359	0.100*	
H6C	0.5836	0.2548	0.9227	0.100*	
C7	0.6312 (3)	0.47267 (19)	0.86320 (12)	0.0650 (5)	
H7A	0.5391	0.5126	0.8832	0.097*	
H7B	0.5903	0.4202	0.8092	0.097*	
H7C	0.7384	0.5261	0.8616	0.097*	
C8	0.8222 (3)	1.47002 (15)	0.57602 (11)	0.0523 (4)	
H8	0.8505	1.4290	0.6089	0.063*	
C9	0.7602 (2)	1.41427 (15)	0.49641 (11)	0.0500 (4)	
H9	0.7461	1.3359	0.4755	0.060*	
C10	0.7169 (2)	1.47433 (14)	0.44473 (10)	0.0446 (4)	
C11	0.7429 (3)	1.59337 (15)	0.48165 (11)	0.0537 (4)	
H11	0.7176	1.6377	0.4508	0.064*	
C12	0.8050 (3)	1.64289 (16)	0.56213 (12)	0.0573 (5)	
H12	0.8210	1.7211	0.5855	0.069*	
C13	0.6263 (4)	1.29817 (19)	0.32983 (13)	0.0759 (6)	
H13A	0.7383	1.2756	0.3343	0.114*	
H13B	0.5807	1.2754	0.2733	0.114*	
H13C	0.5407	1.2625	0.3583	0.114*	
C14	0.6142 (4)	1.4840 (2)	0.31276 (13)	0.0776 (7)	
H14A	0.5189	1.5211	0.3325	0.116*	
H14B	0.5767	1.4324	0.2583	0.116*	
H14C	0.7195	1.5398	0.3126	0.116*	
C15	0.9883 (3)	0.77742 (14)	0.79236 (10)	0.0495 (4)	
C16	0.9024 (2)	0.83033 (12)	0.93876 (10)	0.0403 (3)	
C17	0.9453 (2)	0.87021 (13)	1.02242 (10)	0.0459 (4)	
H17	0.8552	0.8706	1.0571	0.055*	
C18	1.1237 (3)	0.90929 (14)	1.05345 (10)	0.0513 (4)	
H18	1.1552	0.9363	1.1096	0.062*	
C19	1.2561 (2)	0.90851 (14)	1.00132 (12)	0.0526 (4)	
H19	1.3767	0.9347	1.0226	0.063*	
C20	1.2105 (2)	0.86906 (14)	0.91776 (11)	0.0489 (4)	
H20	1.3011	0.8699	0.8834	0.059*	
C21	1.0315 (2)	0.82816 (12)	0.88416 (9)	0.0410 (4)	
C22	0.8177 (2)	1.16928 (13)	0.58212 (10)	0.0456 (4)	
C23	0.9267 (3)	1.12931 (18)	0.52210 (14)	0.0671 (5)	
H23	1.0470	1.1331	0.5369	0.081*	
C24	0.8576 (4)	1.0839 (2)	0.44030 (14)	0.0823 (7)	
H24	0.9322	1.0573	0.4010	0.099*	
C25	0.6807 (4)	1.07774 (18)	0.41684 (13)	0.0755 (7)	
H25	0.6360	1.0473	0.3619	0.091*	
C26	0.5713 (3)	1.11601 (15)	0.47379 (12)	0.0613 (5)	
H26	0.4514	1.1123	0.4583	0.074*	
C27	0.6400 (2)	1.16086 (13)	0.55545 (10)	0.0456 (4)	
C28	0.9034 (2)	1.22402 (14)	0.66958 (11)	0.0503 (4)	
N1	0.9009 (2)	0.57649 (13)	1.15435 (9)	0.0512 (4)	
N2	0.8436 (2)	1.58265 (13)	0.60863 (9)	0.0541 (4)	
N3	0.6704 (2)	0.41060 (13)	0.91725 (8)	0.0511 (4)	
N4	0.6558 (2)	1.42142 (13)	0.36576 (9)	0.0562 (4)	
N5	0.7108 (2)	0.79252 (13)	0.90781 (10)	0.0541 (4)	
N6	0.5164 (2)	1.19959 (14)	0.61527 (12)	0.0626 (4)	
O1	0.6036 (2)	0.76776 (17)	0.95241 (13)	0.0902 (5)	
O2	0.6658 (2)	0.7890 (2)	0.83978 (12)	0.1068 (7)	
O3	0.9533 (3)	0.67136 (11)	0.76561 (8)	0.0799 (5)	
O4	0.9988 (2)	0.84108 (12)	0.75059 (8)	0.0675 (4)	
O5	0.9399 (2)	1.16209 (12)	0.70715 (10)	0.0762 (5)	
O6	0.9380 (3)	1.32923 (11)	0.69509 (9)	0.0787 (5)	
O7	0.5517 (3)	1.2051 (2)	0.68427 (11)	0.1237 (9)	
O8	0.3773 (3)	1.21786 (19)	0.59186 (15)	0.1076 (7)	
O9	0.1837 (2)	1.07159 (15)	0.78781 (11)	0.0777 (4)	
O10	0.7405 (2)	0.93838 (16)	0.68527 (13)	0.0874 (5)	
O11	0.5046 (3)	1.0003 (3)	0.81462 (15)	0.1128 (7)	
H9A	0.141 (6)	1.103 (3)	0.758 (2)	0.169*	
H9B	0.087 (2)	1.020 (2)	0.777 (3)	0.169*	
H10A	0.814 (5)	0.917 (4)	0.709 (2)	0.169*	
H10B	0.803 (5)	1.0021 (18)	0.692 (3)	0.169*	
H11A	0.425 (4)	1.026 (4)	0.798 (3)	0.169*	
H11B	0.566 (5)	0.996 (4)	0.7735 (18)	0.169*	
H1A	0.957 (3)	0.6117 (18)	1.2044 (8)	0.074 (7)*	
H2A	0.887 (3)	1.6140 (18)	0.6610 (7)	0.073 (7)*	

### Atomic displacement parameters (Å^2^)

**Table:**

	*U*^11^	*U*^22^	*U*^33^	*U*^12^	*U*^13^	*U*^23^
C1	0.0534 (10)	0.0527 (9)	0.0438 (9)	0.0111 (7)	−0.0004 (7)	0.0217 (7)
C2	0.0536 (9)	0.0411 (8)	0.0446 (9)	0.0107 (7)	0.0017 (7)	0.0161 (7)
C3	0.0414 (8)	0.0448 (8)	0.0373 (8)	0.0095 (6)	0.0039 (6)	0.0146 (7)
C4	0.0590 (10)	0.0434 (8)	0.0462 (9)	0.0116 (7)	0.0013 (7)	0.0203 (7)
C5	0.0632 (11)	0.0398 (8)	0.0501 (10)	0.0042 (7)	0.0004 (8)	0.0130 (7)
C6	0.0867 (15)	0.0554 (11)	0.0452 (10)	0.0093 (10)	−0.0065 (10)	0.0057 (8)
C7	0.0803 (14)	0.0753 (13)	0.0431 (10)	0.0207 (11)	−0.0041 (9)	0.0240 (9)
C8	0.0630 (11)	0.0519 (10)	0.0439 (9)	0.0088 (8)	−0.0013 (8)	0.0218 (8)
C9	0.0601 (10)	0.0428 (8)	0.0456 (9)	0.0067 (7)	−0.0027 (8)	0.0164 (7)
C10	0.0451 (8)	0.0481 (9)	0.0395 (8)	0.0057 (7)	0.0012 (7)	0.0163 (7)
C11	0.0666 (11)	0.0484 (9)	0.0500 (10)	0.0142 (8)	0.0005 (8)	0.0216 (8)
C12	0.0720 (12)	0.0443 (9)	0.0517 (10)	0.0132 (8)	0.0040 (9)	0.0106 (8)
C13	0.0970 (17)	0.0637 (13)	0.0493 (11)	0.0072 (11)	−0.0159 (11)	0.0035 (9)
C14	0.0997 (17)	0.0870 (16)	0.0473 (11)	0.0099 (13)	−0.0081 (11)	0.0322 (11)
C15	0.0684 (11)	0.0442 (9)	0.0377 (8)	0.0162 (8)	0.0020 (8)	0.0144 (7)
C16	0.0472 (8)	0.0311 (7)	0.0436 (8)	0.0097 (6)	0.0005 (7)	0.0139 (6)
C17	0.0623 (10)	0.0373 (8)	0.0416 (9)	0.0152 (7)	0.0094 (7)	0.0147 (6)
C18	0.0741 (12)	0.0386 (8)	0.0375 (8)	0.0126 (8)	−0.0067 (8)	0.0090 (7)
C19	0.0512 (10)	0.0431 (9)	0.0580 (11)	0.0075 (7)	−0.0093 (8)	0.0125 (8)
C20	0.0505 (9)	0.0450 (9)	0.0516 (10)	0.0120 (7)	0.0071 (7)	0.0154 (7)
C21	0.0544 (9)	0.0314 (7)	0.0381 (8)	0.0117 (6)	0.0023 (7)	0.0114 (6)
C22	0.0553 (9)	0.0356 (8)	0.0429 (9)	0.0062 (7)	−0.0054 (7)	0.0125 (7)
C23	0.0645 (12)	0.0624 (12)	0.0701 (13)	0.0136 (10)	0.0101 (10)	0.0157 (10)
C24	0.117 (2)	0.0663 (14)	0.0559 (13)	0.0153 (13)	0.0275 (13)	0.0100 (11)
C25	0.118 (2)	0.0532 (11)	0.0406 (10)	−0.0022 (12)	−0.0100 (12)	0.0109 (9)
C26	0.0772 (13)	0.0440 (9)	0.0540 (11)	−0.0037 (9)	−0.0243 (10)	0.0182 (8)
C27	0.0566 (9)	0.0342 (7)	0.0428 (9)	0.0042 (7)	−0.0085 (7)	0.0135 (6)
C28	0.0553 (10)	0.0452 (9)	0.0476 (9)	0.0046 (7)	−0.0131 (8)	0.0175 (7)
N1	0.0546 (8)	0.0524 (8)	0.0401 (8)	0.0022 (7)	−0.0065 (6)	0.0134 (6)
N2	0.0665 (10)	0.0548 (9)	0.0360 (8)	0.0098 (7)	0.0001 (7)	0.0107 (7)
N3	0.0630 (9)	0.0512 (8)	0.0380 (7)	0.0131 (7)	−0.0022 (6)	0.0137 (6)
N4	0.0667 (10)	0.0580 (9)	0.0404 (8)	0.0069 (7)	−0.0061 (7)	0.0172 (7)
N5	0.0502 (8)	0.0475 (8)	0.0660 (10)	0.0103 (6)	−0.0011 (7)	0.0220 (7)
N6	0.0550 (9)	0.0560 (9)	0.0715 (11)	0.0113 (7)	−0.0042 (8)	0.0156 (8)
O1	0.0539 (9)	0.1180 (14)	0.1136 (14)	0.0119 (9)	0.0183 (9)	0.0619 (12)
O2	0.0651 (10)	0.172 (2)	0.0772 (12)	0.0044 (11)	−0.0220 (9)	0.0492 (13)
O3	0.1489 (16)	0.0448 (7)	0.0383 (7)	0.0178 (8)	−0.0128 (8)	0.0077 (6)
O4	0.1011 (11)	0.0583 (8)	0.0494 (7)	0.0185 (7)	0.0010 (7)	0.0271 (6)
O5	0.0974 (11)	0.0614 (8)	0.0711 (9)	0.0069 (8)	−0.0274 (8)	0.0346 (7)
O6	0.1240 (13)	0.0446 (7)	0.0532 (8)	0.0023 (7)	−0.0385 (8)	0.0119 (6)
O7	0.0940 (14)	0.211 (3)	0.0547 (11)	0.0546 (15)	0.0089 (9)	0.0146 (13)
O8	0.0775 (12)	0.1227 (16)	0.1424 (18)	0.0515 (11)	0.0068 (12)	0.0535 (14)
O9	0.0827 (11)	0.0792 (11)	0.0707 (10)	0.0185 (8)	−0.0039 (8)	0.0252 (8)
O10	0.0776 (11)	0.0778 (11)	0.1062 (14)	0.0037 (9)	−0.0192 (10)	0.0413 (10)
O11	0.0952 (15)	0.153 (2)	0.0994 (16)	0.0310 (14)	0.0013 (12)	0.0531 (15)

### Geometric parameters (Å, º)

**Table:**

C1—N1	1.341 (2)	C15—O3	1.250 (2)
C1—C2	1.355 (2)	C15—C21	1.514 (2)
C1—H1	0.9300	C16—C17	1.381 (2)
C2—C3	1.416 (2)	C16—C21	1.390 (2)
C2—H2	0.9300	C16—N5	1.464 (2)
C3—N3	1.339 (2)	C17—C18	1.374 (3)
C3—C4	1.417 (2)	C17—H17	0.9300
C4—C5	1.354 (2)	C18—C19	1.380 (3)
C4—H4	0.9300	C18—H18	0.9300
C5—N1	1.338 (2)	C19—C20	1.382 (3)
C5—H5	0.9300	C19—H19	0.9300
C6—N3	1.456 (2)	C20—C21	1.391 (2)
C6—H6A	0.9600	C20—H20	0.9300
C6—H6B	0.9600	C22—C27	1.385 (2)
C6—H6C	0.9600	C22—C23	1.392 (3)
C7—N3	1.459 (2)	C22—C28	1.514 (2)
C7—H7A	0.9600	C23—C24	1.390 (3)
C7—H7B	0.9600	C23—H23	0.9300
C7—H7C	0.9600	C24—C25	1.372 (4)
C8—N2	1.339 (2)	C24—H24	0.9300
C8—C9	1.355 (2)	C25—C26	1.354 (3)
C8—H8	0.9300	C25—H25	0.9300
C9—C10	1.415 (2)	C26—C27	1.387 (2)
C9—H9	0.9300	C26—H26	0.9300
C10—N4	1.339 (2)	C27—N6	1.465 (3)
C10—C11	1.416 (2)	C28—O5	1.232 (2)
C11—C12	1.360 (3)	C28—O6	1.239 (2)
C11—H11	0.9300	N1—H1A	0.886 (10)
C12—N2	1.335 (2)	N2—H2A	0.890 (10)
C12—H12	0.9300	N5—O2	1.201 (2)
C13—N4	1.461 (3)	N5—O1	1.210 (2)
C13—H13A	0.9600	N6—O7	1.193 (2)
C13—H13B	0.9600	N6—O8	1.211 (2)
C13—H13C	0.9600	O9—H9A	0.833 (10)
C14—N4	1.455 (3)	O9—H9B	0.850 (10)
C14—H14A	0.9600	O10—H10A	0.823 (10)
C14—H14B	0.9600	O10—H10B	0.824 (10)
C14—H14C	0.9600	O11—H11A	0.826 (10)
C15—O4	1.238 (2)	O11—H11B	0.861 (10)
			
N1—C1—C2	121.77 (15)	C21—C16—N5	119.65 (15)
N1—C1—H1	119.1	C18—C17—C16	118.75 (16)
C2—C1—H1	119.1	C18—C17—H17	120.6
C1—C2—C3	120.51 (15)	C16—C17—H17	120.6
C1—C2—H2	119.7	C17—C18—C19	120.07 (16)
C3—C2—H2	119.7	C17—C18—H18	120.0
N3—C3—C2	121.99 (15)	C19—C18—H18	120.0
N3—C3—C4	122.39 (15)	C18—C19—C20	120.39 (17)
C2—C3—C4	115.62 (14)	C18—C19—H19	119.8
C5—C4—C3	120.42 (15)	C20—C19—H19	119.8
C5—C4—H4	119.8	C19—C20—C21	121.14 (16)
C3—C4—H4	119.8	C19—C20—H20	119.4
N1—C5—C4	121.97 (16)	C21—C20—H20	119.4
N1—C5—H5	119.0	C16—C21—C20	116.66 (15)
C4—C5—H5	119.0	C16—C21—C15	123.84 (15)
N3—C6—H6A	109.5	C20—C21—C15	119.37 (15)
N3—C6—H6B	109.5	C27—C22—C23	116.35 (17)
H6A—C6—H6B	109.5	C27—C22—C28	125.37 (16)
N3—C6—H6C	109.5	C23—C22—C28	118.17 (17)
H6A—C6—H6C	109.5	C24—C23—C22	120.7 (2)
H6B—C6—H6C	109.5	C24—C23—H23	119.7
N3—C7—H7A	109.5	C22—C23—H23	119.7
N3—C7—H7B	109.5	C25—C24—C23	120.8 (2)
H7A—C7—H7B	109.5	C25—C24—H24	119.6
N3—C7—H7C	109.5	C23—C24—H24	119.6
H7A—C7—H7C	109.5	C26—C25—C24	119.88 (19)
H7B—C7—H7C	109.5	C26—C25—H25	120.1
N2—C8—C9	121.68 (16)	C24—C25—H25	120.1
N2—C8—H8	119.2	C25—C26—C27	119.3 (2)
C9—C8—H8	119.2	C25—C26—H26	120.3
C8—C9—C10	120.53 (16)	C27—C26—H26	120.3
C8—C9—H9	119.7	C22—C27—C26	122.93 (18)
C10—C9—H9	119.7	C22—C27—N6	119.45 (15)
N4—C10—C9	121.79 (16)	C26—C27—N6	117.62 (17)
N4—C10—C11	122.36 (15)	O5—C28—O6	126.17 (16)
C9—C10—C11	115.85 (15)	O5—C28—C22	118.31 (15)
C12—C11—C10	120.03 (16)	O6—C28—C22	115.42 (14)
C12—C11—H11	120.0	C5—N1—C1	119.69 (15)
C10—C11—H11	120.0	C5—N1—H1A	120.8 (16)
N2—C12—C11	122.02 (17)	C1—N1—H1A	119.5 (15)
N2—C12—H12	119.0	C12—N2—C8	119.89 (15)
C11—C12—H12	119.0	C12—N2—H2A	123.1 (15)
N4—C13—H13A	109.5	C8—N2—H2A	117.0 (15)
N4—C13—H13B	109.5	C3—N3—C6	121.71 (15)
H13A—C13—H13B	109.5	C3—N3—C7	121.54 (15)
N4—C13—H13C	109.5	C6—N3—C7	116.73 (15)
H13A—C13—H13C	109.5	C10—N4—C14	121.44 (16)
H13B—C13—H13C	109.5	C10—N4—C13	121.11 (15)
N4—C14—H14A	109.5	C14—N4—C13	117.45 (16)
N4—C14—H14B	109.5	O2—N5—O1	122.49 (18)
H14A—C14—H14B	109.5	O2—N5—C16	118.90 (16)
N4—C14—H14C	109.5	O1—N5—C16	118.60 (17)
H14A—C14—H14C	109.5	O7—N6—O8	122.5 (2)
H14B—C14—H14C	109.5	O7—N6—C27	118.58 (18)
O4—C15—O3	125.90 (16)	O8—N6—C27	118.8 (2)
O4—C15—C21	119.44 (15)	H9A—O9—H9B	91 (4)
O3—C15—C21	114.55 (14)	H10A—O10—H10B	96 (4)
C17—C16—C21	123.00 (15)	H11A—O11—H11B	96 (4)
C17—C16—N5	117.32 (15)		
			
N1—C1—C2—C3	0.9 (3)	C24—C25—C26—C27	−0.1 (3)
C1—C2—C3—N3	178.43 (16)	C23—C22—C27—C26	−0.3 (2)
C1—C2—C3—C4	−1.8 (2)	C28—C22—C27—C26	175.91 (15)
N3—C3—C4—C5	−178.69 (17)	C23—C22—C27—N6	179.05 (16)
C2—C3—C4—C5	1.6 (2)	C28—C22—C27—N6	−4.7 (2)
C3—C4—C5—N1	−0.3 (3)	C25—C26—C27—C22	0.4 (3)
N2—C8—C9—C10	0.4 (3)	C25—C26—C27—N6	−179.00 (17)
C8—C9—C10—N4	179.85 (17)	C27—C22—C28—O5	108.3 (2)
C8—C9—C10—C11	0.1 (3)	C23—C22—C28—O5	−75.5 (2)
N4—C10—C11—C12	179.88 (18)	C27—C22—C28—O6	−75.0 (2)
C9—C10—C11—C12	−0.4 (3)	C23—C22—C28—O6	101.1 (2)
C10—C11—C12—N2	0.2 (3)	C4—C5—N1—C1	−0.7 (3)
C21—C16—C17—C18	−0.2 (2)	C2—C1—N1—C5	0.4 (3)
N5—C16—C17—C18	−178.21 (13)	C11—C12—N2—C8	0.3 (3)
C16—C17—C18—C19	0.2 (2)	C9—C8—N2—C12	−0.6 (3)
C17—C18—C19—C20	0.3 (3)	C2—C3—N3—C6	3.1 (3)
C18—C19—C20—C21	−0.8 (3)	C4—C3—N3—C6	−176.64 (17)
C17—C16—C21—C20	−0.2 (2)	C2—C3—N3—C7	−175.62 (17)
N5—C16—C21—C20	177.74 (13)	C4—C3—N3—C7	4.6 (3)
C17—C16—C21—C15	175.61 (14)	C9—C10—N4—C14	−179.02 (19)
N5—C16—C21—C15	−6.5 (2)	C11—C10—N4—C14	0.7 (3)
C19—C20—C21—C16	0.7 (2)	C9—C10—N4—C13	1.7 (3)
C19—C20—C21—C15	−175.32 (15)	C11—C10—N4—C13	−178.59 (19)
O4—C15—C21—C16	107.6 (2)	C17—C16—N5—O2	158.56 (19)
O3—C15—C21—C16	−75.9 (2)	C21—C16—N5—O2	−19.5 (3)
O4—C15—C21—C20	−76.7 (2)	C17—C16—N5—O1	−20.3 (2)
O3—C15—C21—C20	99.8 (2)	C21—C16—N5—O1	161.69 (17)
C27—C22—C23—C24	0.0 (3)	C22—C27—N6—O7	−22.2 (3)
C28—C22—C23—C24	−176.52 (18)	C26—C27—N6—O7	157.2 (2)
C22—C23—C24—C25	0.2 (3)	C22—C27—N6—O8	162.33 (19)
C23—C24—C25—C26	−0.2 (3)	C26—C27—N6—O8	−18.2 (3)

### Hydrogen-bond geometry (Å, º)

Cg1, Cg3 and Cg4 are the centroids of rings N1/C1–C5, C16–C21, and C22–C27, respectively.

**Table:**

*D*—H···*A*	*D*—H	H···*A*	*D*···*A*	*D*—H···*A*
N1—H1*A*···O6^i^	0.89 (2)	1.75 (2)	2.639 (2)	175 (2)
O9—H9*A*···O5^ii^	0.84 (4)	2.11 (4)	2.896 (2)	156 (4)
O10—H10*A*···O4	0.82 (4)	2.08 (4)	2.892 (2)	170 (4)
O10—H10*B*···O5	0.82 (3)	2.02 (3)	2.847 (3)	176 (4)
O11—H11*A*···O9	0.83 (4)	2.05 (3)	2.841 (3)	160 (4)
O11—H11*B*···O10	0.86 (4)	2.12 (4)	2.943 (3)	160 (4)
C1—H1···O3^iii^	0.93	2.43	3.348 (2)	169
C4—H4···O1	0.93	2.57	3.474 (3)	164
C6—H6*B*···O7^iv^	0.96	2.51	3.258 (3)	135
C8—H8···O6	0.93	2.40	3.325 (3)	177
C23—H23···O8^v^	0.93	2.55	3.434 (3)	160
C2—H2···*Cg*3^iii^	0.93	2.96	3.7481 (19)	144
C7—H7*A*···*Cg*1^vi^	0.93	2.85	3.661 (2)	142
C9—H9···*Cg*4	0.93	2.86	3.702 (2)	151

Symmetry codes: (i) −*x*+2, −*y*+2, −*z*+2; (ii) *x*−1, *y*, *z*; (iii) −*x*+2, −*y*+1, −*z*+2; (iv) *x*, *y*−1, *z*; (v) *x*+1, *y*, *z*; (vi) −*x*+1, −*y*+1, −*z*+2.
